# Supplementary material for: Red2Flpe-SCON: a versatile, multicolor strategy for generating mosaic conditional knockout mice
Source: Nat Commun. 2024 Jun 11;15:4963. doi: 10.1038/s41467-024-49382-y (PMC11166929; doi:10.1038/s41467-024-49382-y)
Supplement: Supplementary file 1 — Supplementary Information [file 41467_2024_49382_MOESM1_ESM.pdf]

Supplementary Information

Red2Flpe-SCON: A Versatile, Multicolor Strategy for Generating Mosaic Conditional Knockout Mice

Szu-Hsien Sam Wu et al.

Correspondence should be addressed to G.C. (gabriele.colozza@imba.oeaw.ac.at) and B.-K.K. (koobk@ibs.re.kr)

This file includes:

Supplementary Figures 1-7.

Supplementary Table 1.

Supplementary Table 2.

Supplementary Fig. 1

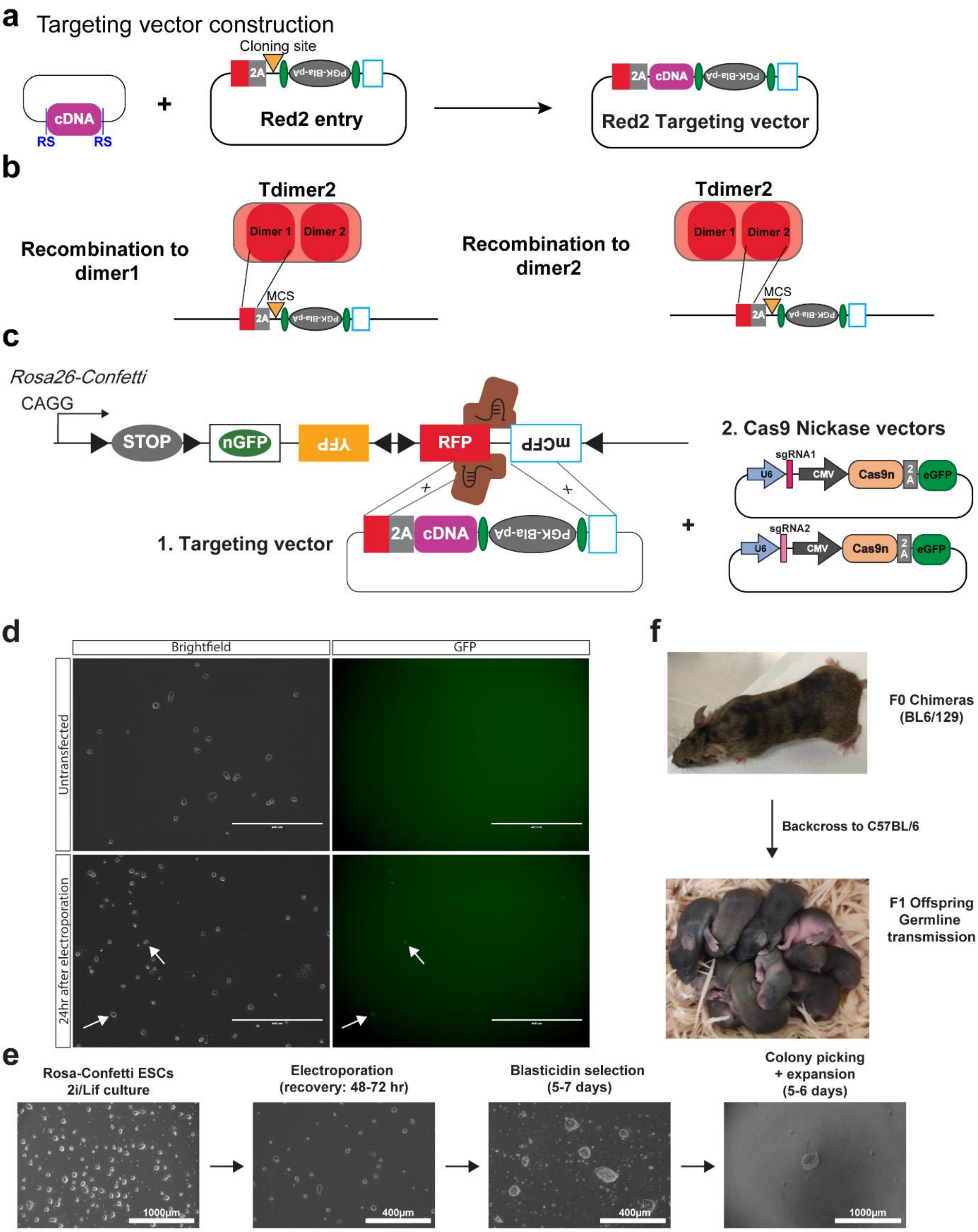

Supplementary Fig. 1 | Pipeline for generating new Red2cDNA mouse lines. a, The Red2 targeting vector consists of homology arms that correspond to parts of the RFP and mCFP sequences, with a PGK-blasticidin-pA cassette in a reversed orientation, flanked by FRT sites (in green), and a cloning site for any cDNA sequence downstream of a P2A peptide. **b,** The left homology arm of the Red2 targeting vector can be recombined to either one of the RFP (tdimer2) dimer sequences. **c,** Targeting onto the confetti allele is achieved with a pair of Cas9 nickase vectors, that contain gRNA targeted to RFP at the 3' terminus. **d,** Successful delivery of the targeting components can be checked 24hrs post-electroporation, where eGFP expression from the Cas9 nickase vectors can be observed in a small subset of cells. **e,** Steps for ESC targeting (which requires 2.5-3 weeks), including initial expansion of ESCs, electroporation, blasticidin selection, colony picking, and expansion. **f,** Chimeric mice are generated after the correct targeting clones (B6/129F1 background) have been injected into a developing blastocyst (C57BL/6), and backcrossing to confirm germline transmission (agouti pups) has been completed. Experiment in **d-f** was performed using different Confetti ESC clones for multiple times ($n > 5$).

Supplementary Fig. 2

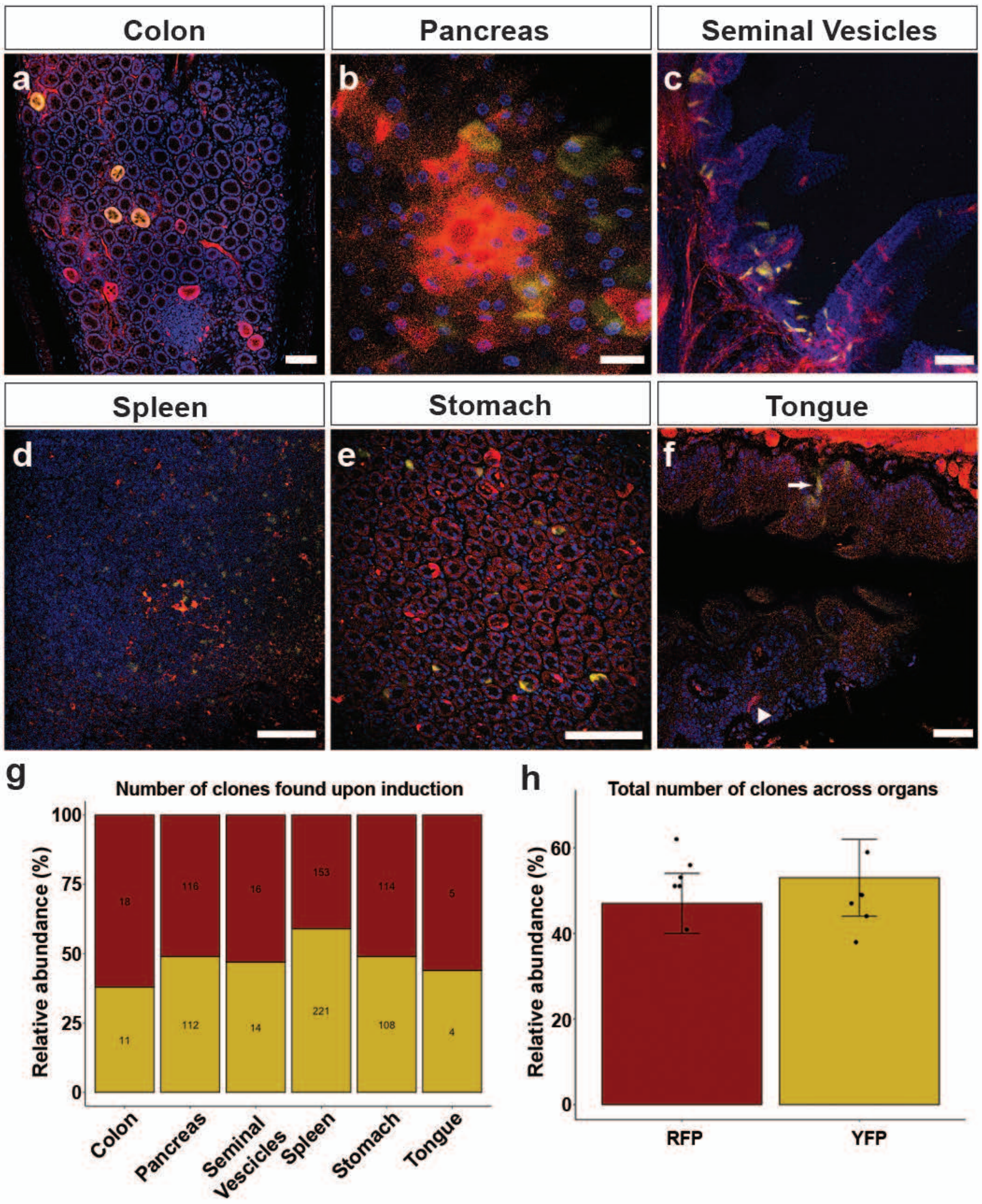

Supplementary Fig. 2 | Usability of Red2Flpe in multiple organs. a-f, Fluorescence microscopy showing efficient YFP+ and RFP+ clone formation in the organs indicated. Scale bars: 30 µm (a-e)**;** 20 µm (f). YFP+ and RFP+ cells are marked by triangles and arrows, respectively. **g** and **h**, Quantification and relative abundance of YFP+ and RFP+ clones in individual organs and in total, respectively. The total number of clones counted is indicated in each bar (p value = 0.8587). Dots represent relative abundance of the different organs. Experiment in **a-f** was performed in two different mice of Rosa-CreERT2; Red2Flpe to examine the induction in different organs. Source Data relevant to this Figure are provided with this paper in Source Data file.

Supplementary Fig. 3

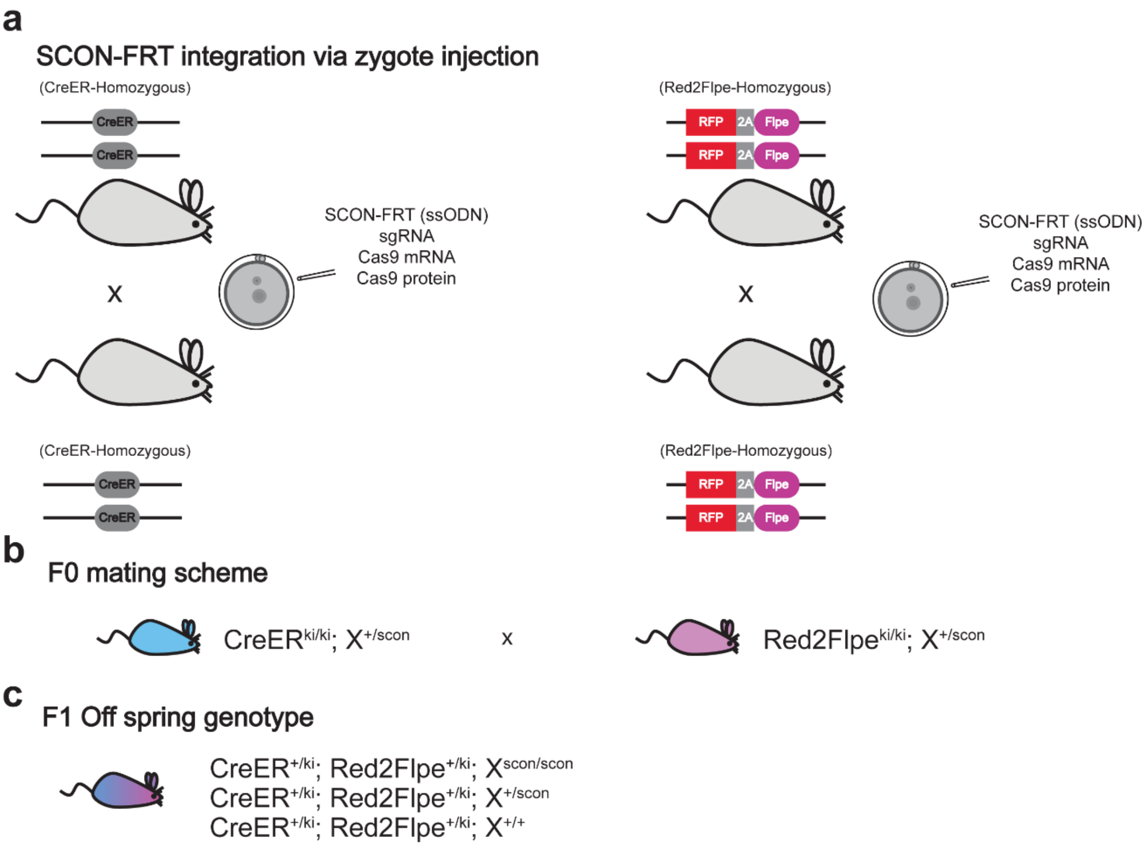

Supplementary Fig. 3 | Generation of mosaic conditional mice via zygote injection. a, Parallel zygote targeting of SCON-FRT to target the gene in the desired CreER and Red2Flpe homozygous mice. **b,** All offspring contain homozygous CreER and Red2Flpe. Pups containing precise SCON-FRT integration in the gene of interest (gene X) are bred to obtain experimental cohorts and confirm germline transmission of X^{scon}. **c,** There is a ¼ chance of obtaining experimental cohorts containing CreER (heterozygous), Red2Flpe (heterozygous) and SCON-FRT (homozygous).

Supplementary Fig. 4

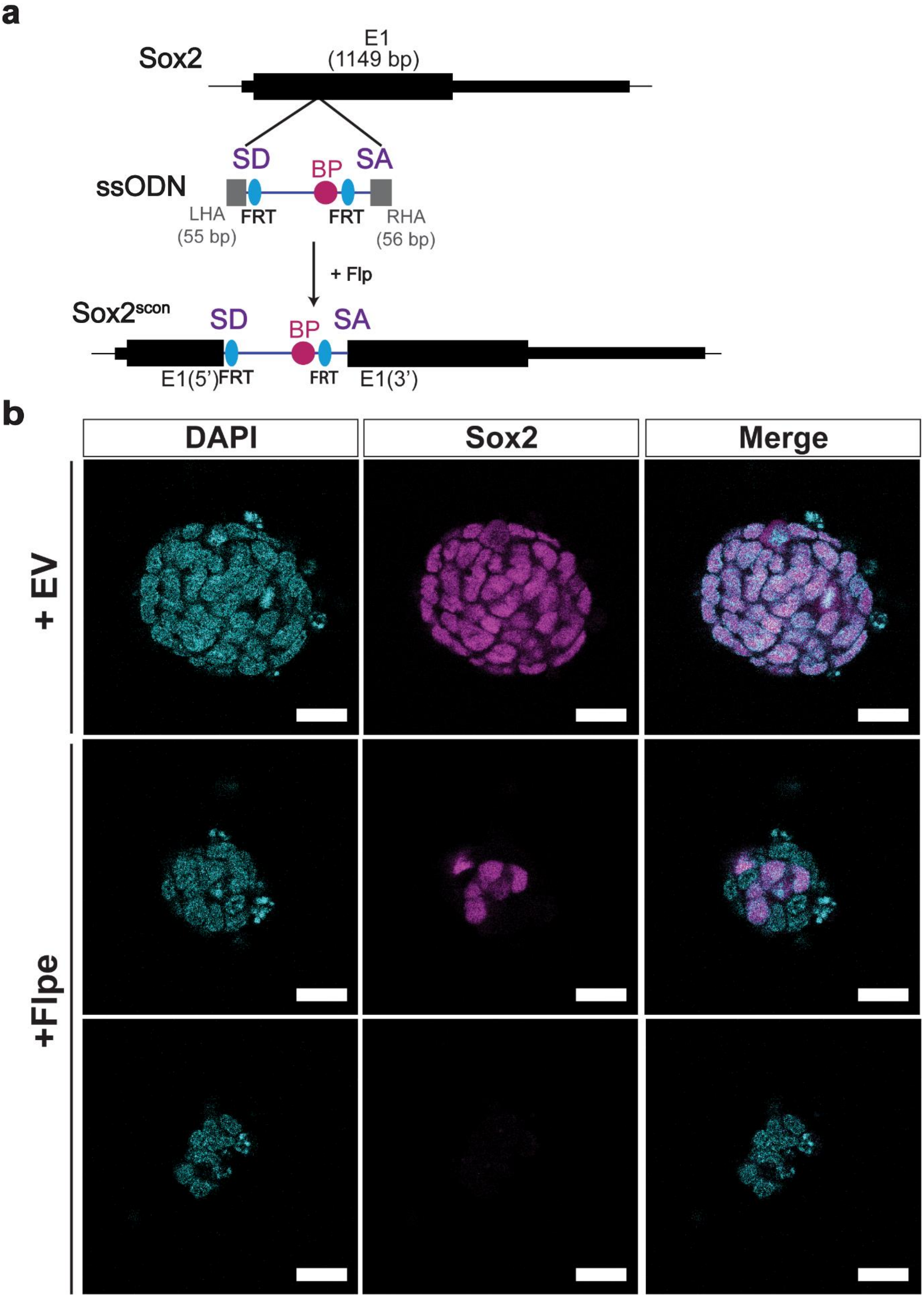

Supplementary Fig. 4 | Sox2-SCON-FRT: A new cKO mouse line that functions as expected. a, Design of the Sox2-SCON-FRT allele. The synthesized ssODN of the 189bp long SCON-FRT cassette, with homology arms of 55–56 bp, is inserted into the Sox2 allele via zygote injection. **b,** Images of Sox2-SCON-FRT homozygous mouse ESCs 48 hours after transfection with either the Flpe-expressing plasmid or an empty vector control. Scale bar, 20 μm. The experiment shown in **b** was performed in 2 different $Sox2^{scon/scon}$ ESC clones for two times, with similar results.

Supplementary Fig. 5

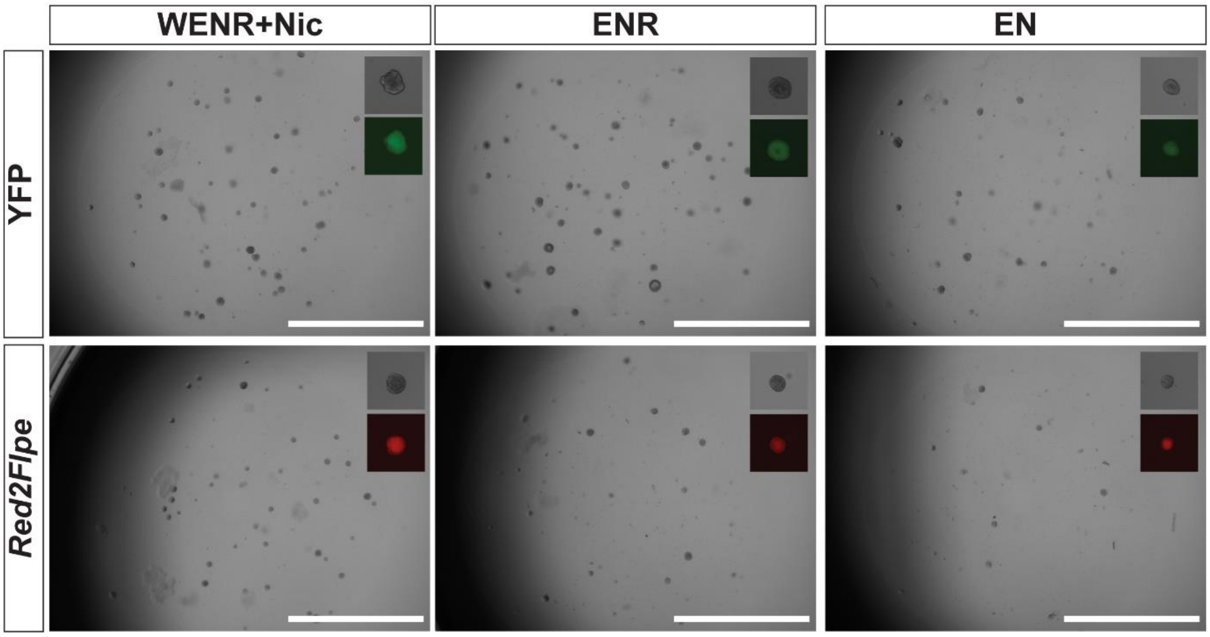

Supplementary Fig. 5 | Sox2 knockout esophageal cells show reduced self-renewal and organoid forming efficiency. RFP+ and YFP+ esophageal organoids of *Rosa*$^{CreERT2/Red2Flpe}$*;* *Sox2*$^{scon/scon}$, cultured in WENR+Nic (Wnt3a-condition medium, EGF, Noggin, R-spondin1 and nicotinamide), ENR (EGF, Noggin and R-spondin1) or EN (EGF and Noggin). Scale bar, 2 mm. The establishment of esophageal organoids was repeated from 2 different mice and subjected to 4OH-tamoxifen induction and different growth media cultures, the morphology and growth across different lines were similar.

Supplementary Fig. 6

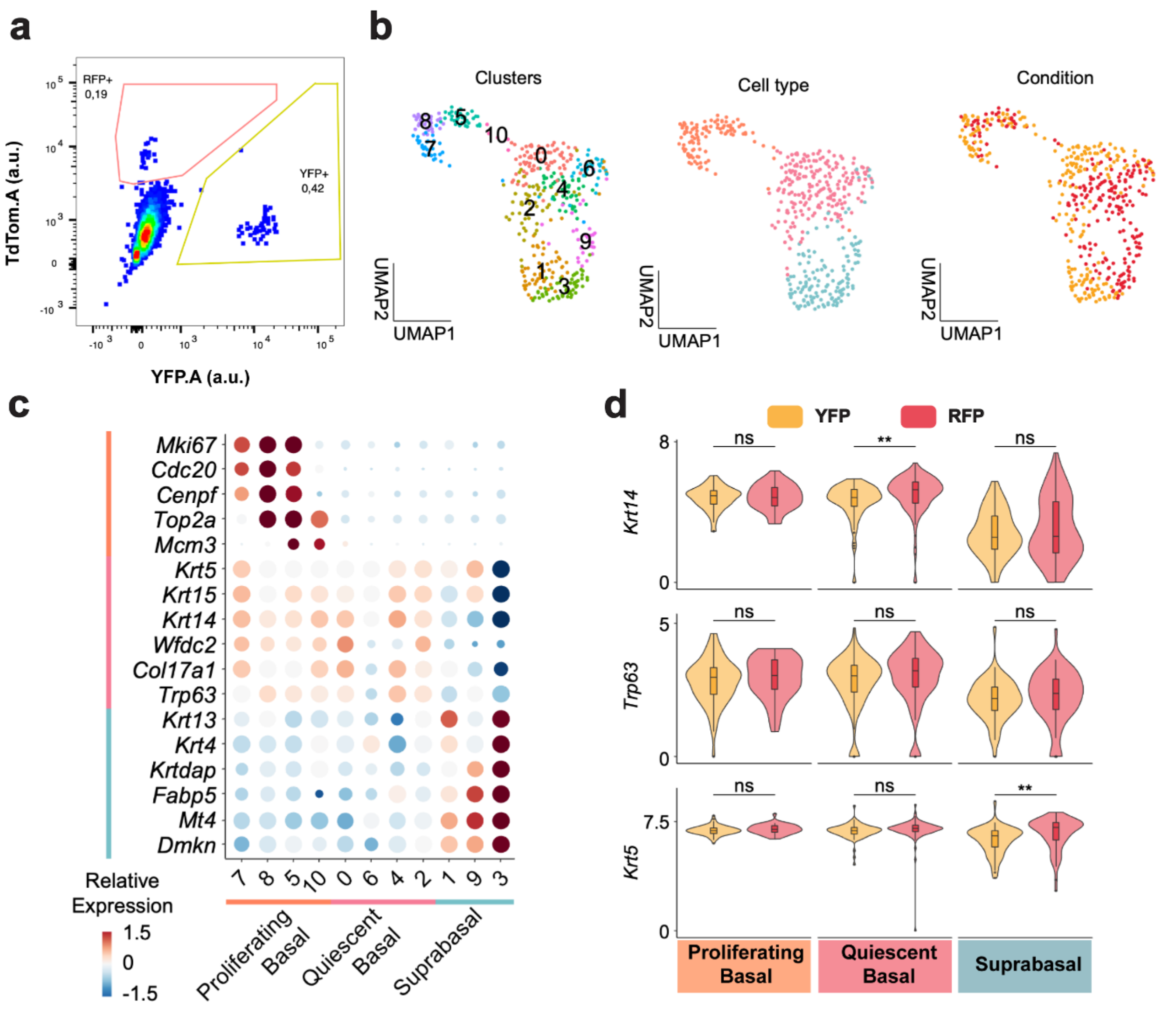

Supplementary Fig. 6 | scRNA seq on Red2Flpe Sox2 mutant mice. a, FACS sorting of WT (YFP+) and Sox2 KO (RFP+) clones for single-cell RNA sequencing (scRNA-seq). **b,** UMAP plots of mouse esophageal cells showing clusters (left), cell types (middle), and genotypic conditions (right). **c,** Dot plots for cell type annotation. For each cluster, the average expression levels of the corresponding gene are shown color-coded according to the scale on the bottom, with a gradient from blue (minimum expression) to red (maximum expression). The size of the dots indicates the fraction of cells showing expression of the corresponding genes. **d,** Violin plot showing expression comparison of the indicated markers between WT (YFP+) and Sox2 KO (RFP+) clones. Violin and box plots indicate the distribution of data where minimum, maximum and central 50% of values are presented. p value of ** < 0.01 calculated by two-tailed unpaired Student's *t*-test. For the scRNA-seq experiment, 3 mice of *Rosa-CreERT2; Red2Flpe; Sox2*$^{scon/scon}$ induced for one month were used for sorting induced esophageal cells. Source Data relevant to this Figure are provided with this paper in Source Data file.

Supplementary Fig. 7

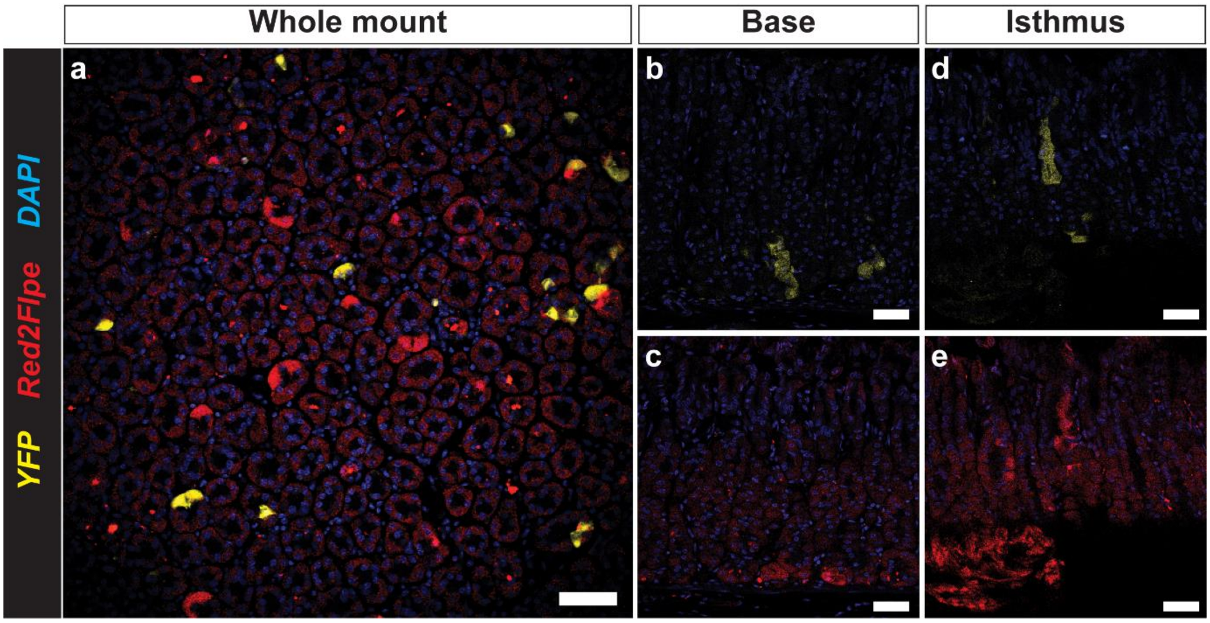

Supplementary Fig. 7 | Sox2 mosaic knockout in the adult stomach 4 weeks after induction. a, Wholemount of the corpus epithelium reveals clonality progression of the glands. Scale bar, 40 µm. **b** and **c,** Basal clones of YFP+ and RFP+ cells. Scale bar, 20 µm. **d** and **e**, Isthmus clones of YFP+ and RFP+ cells. Scale bar, 20 µm.

Supplementary Table 1. Comparison of different in vivo mosaic genetics systems.

	**Single color labeling**	**Multicolor labeling**		
Mosaic system	**Dual Recombinase**	**MADM**	**Dual-ifgMosaics**	**Red2cDNA/ Red2Flpe-SCON**
References	Lao *et al*. (2012) *Cell Rep*.[1]<br>Thorsen *et al*. (2021) *Disease Models & Mech*.[2]	Zong *et al*. (2005) *Cell* [3]<br>Conteras *et al*. (2021) *Cell Rep*. [4]	Pontes-Quero *et al*. (2017) *Cell* [5]	Yum *et al*. (2021) *Nature* [6]<br>Wu *et al*. (Current study)
Type of mosaicism	i) Overexpression<br>ii) Conditional knockout mutants	Loss of Heterozygosity	Overexpression	i) Overexpression<br>ii) Conditional knockout mutants
Mechanism	Short range recombination	Interchromosomal recombination	Short range recombination	Short range recombination
Allele construction	Breeding (existing) mouselines	Chromosomal rearrangement through series of breeding	BAC Cloning and insertion of multicolor reporters in mouse ES cells	CRISPR-mediated targeting in mouse ES cells
Labelling ratio	N/A	1:1	Variable between lines	2:2:1<br>(Yellow : Red : Cyan)
Mosaic quantifiability	+/-	+++	+	+++
Tunability	1-100%	0-5%<br>(from cycling cells)	0-33%	0-40%

Main advantages	Utilize existing mouse lines	i) Labelled cells originate from the same mother cell.<br>ii) Allows the investigation of genetic imprinting effects.	i) Combination of multiple fluorescent proteins are used.<br>ii) Efficient generation of new lines.	i) Quantitative and tunable mosaic analysis.<br>ii) Efficient generation of new lines.<br>iii) Possibility for multiplxed knockout and/ or overexpression
Main disadvantages	i) Incoherent genotype and reporter link (thus requires careful confirmation of true knockout).<br>ii) Lack of wild type cell lineage information.	i) Not tunable<br>ii) Not applicable in haploinsufficient genes<br>iii) Works only in strong Cre expressing lines and proliferative cells.	i) Unequal initial labeling.<br>ii) Only permits ectopic gene overexpression	Requires making of FRT-based cKO mice

Supplementary Table 2. Sequences of oligos and primers.

	**Sequence**
**Nicking sgRNA pair used for generating Red2cDNA**	CGGCCAATTCCTACAGGAAC
	TAGAGTCGCGGCTAGATAAT
**VilCreER^{T2} (Expected size: 280bp)**	
Vil_Fwd	CAAGCCTGGCTCGACGGCC
Vil_Rev	CGCGAACATCTTCAGGTTCT
**Red2Flpe 5' integration (Expected size: 2542 bp)**	
Red2Flpe_FWD	AGACGTTGTGGCTGTTGTAGTTGTACTC
Red2Flpe_REV	CCCAGATGCTTTCACCCTCACTTAGAAG
**Red2Flpe 3' integration (Expected size: 2106 bp)**	
Red2cDNA_FWD	TAGTCTTCAGAGATGGGGATGCTGTTGA
Red2cDNA_REV	GCGCGATTCGAGATAACTTCGTATAGCA
**Apc$^{FRT/FRT}$ (WT = 251 bp, Knock-in = 291 bp)**	
ApcFRT_FWD	CTGCAGATCTCTCCAAGGCC
ApcFRT_REV	CAAGTGACTGGCAGATGGGT
**Rosa26-RCE:FRT (WT = 96 bp, Knock-in = 140 bp)**	
RCE-FRT_FWD	CTTCCCTCGTGATCTGCAAC
RCE-FRT_REV	GTTATGTAACGCGGAACTCCA
Rosa_REV	CAGGACAACGCCCACACA
**Rosa26-CreERT2 (Expected size: 102 bp)**	
Cre_FWD	GCGGTCTGGCAGTAAAAACTATC
Cre_Rev	GTGAAACAGCATTGCTGTCACTT
**Sox2$^{scon/scon}$ (WT = 206bp, Knock-in = 395bp)**	
Sox2_F1	GGGCGCGGAGTGGAAACTTTTGTCCG
Sox2_R1	CAACCCCGCTCGCCATGCTGTTCC

Supplementary References

1. Lao, Z., Raju, G.P., Bai, C.B. & Joyner, A.L. MASTR: a technique for mosaic mutant analysis with spatial and temporal control of recombination using conditional floxed alleles in mice. Cell Rep 2, 386-396 (2012).
2. Thorsen, A.S. et al. Heterogeneity in clone dynamics within and adjacent to intestinal tumours identified by Dre-mediated lineage tracing. Dis Model Mech 14 (2021).
3. Zong, H., Espinosa, J.S., Su, H.H., Muzumdar, M.D. & Luo, L. Mosaic analysis with double markers in mice. Cell 121, 479-492 (2005).
4. Contreras, X. et al. A genome-wide library of MADM mice for single-cell genetic mosaic analysis. Cell Rep 35, 109274 (2021).
5. Pontes-Quero, S. et al. Dual ifgMosaic: A Versatile Method for Multispectral and Combinatorial Mosaic Gene-Function Analysis. Cell 170, 800-814 e818 (2017).
6. Yum, M.K. et al. Tracing oncogene-driven remodelling of the intestinal stem cell niche. Nature 594, 442-447 (2021).
