# Supplementary material for: Red2Flpe-SCON: a versatile, multicolor strategy for generating mosaic conditional knockout mice
Source: Nat Commun. 2024 Jun 11;15:4963. doi: 10.1038/s41467-024-49382-y (PMC11166929; doi:10.1038/s41467-024-49382-y)
Supplement: Supplementary file 3 — Description of Additional Supplementary Files [file 41467_2024_49382_MOESM3_ESM.pdf]

Description of Additional Supplementary Files

Supplementary data 1. Sample information plate #1 for the scRNAseq run. 384-well plate #1 information of the scRNAseq experiment, including the well coordinate, sampled mouse number, the corresponding barcode sequence and fluorescent label from FACS.

Supplementary data 2. Sample information plate #2 for the scRNAseq run. 384-well plate #2 information of the scRNAseq experiment, including the well coordinate, sampled mouse number, the corresponding barcode sequence and fluorescent label from FACS.
