# Supplementary material for: Red2Flpe-SCON: a versatile, multicolor strategy for generating mosaic conditional knockout mice
Source: Nat Commun. 2024 Jun 11;15:4963. doi: 10.1038/s41467-024-49382-y (PMC11166929; doi:10.1038/s41467-024-49382-y)
Supplement: Supplementary file 6 — Reporting Summary [file 41467_2024_49382_MOESM6_ESM.pdf]

nature portfolio

Corresponding author(s): Gabriele Colozza, Bon-Kyoung Koo

Last updated by author(s): May 8, 2024

Reporting Summary

Nature Portfolio wishes to improve the reproducibility of the work that we publish. This form provides structure for consistency and transparency in reporting. For further information on Nature Portfolio policies, see our Editorial Policies and the Editorial Policy Checklist.

Statistics

For all statistical analyses, confirm that the following items are present in the figure legend, table legend, main text, or Methods section.

n/a	Confirmed	
☐	☒	The exact sample size (*n*) for each experimental group/condition, given as a discrete number and unit of measurement
☐	☒	A statement on whether measurements were taken from distinct samples or whether the same sample was measured repeatedly
☐	☒	The statistical test(s) used AND whether they are one- or two-sided<br>*Only common tests should be described solely by name; describe more complex techniques in the Methods section.*
☒	☐	A description of all covariates tested
☒	☐	A description of any assumptions or corrections, such as tests of normality and adjustment for multiple comparisons
☐	☒	A full description of the statistical parameters including central tendency (e.g. means) or other basic estimates (e.g. regression coefficient) AND variation (e.g. standard deviation) or associated estimates of uncertainty (e.g. confidence intervals)
☐	☒	For null hypothesis testing, the test statistic (e.g. *F*, *t*, *r*) with confidence intervals, effect sizes, degrees of freedom and *P* value noted<br>*Give P values as exact values whenever suitable.*
☒	☐	For Bayesian analysis, information on the choice of priors and Markov chain Monte Carlo settings
☒	☐	For hierarchical and complex designs, identification of the appropriate level for tests and full reporting of outcomes
☒	☐	Estimates of effect sizes (e.g. Cohen's *d*, Pearson's *r*), indicating how they were calculated

Our web collection on statistics for biologists contains articles on many of the points above.

Software and code

Policy information about availability of computer code

Data collection	Confocal images were obtained using a Leica Sp8 multiphoton microscope and displayed with the LAS software (Leica). Flow cytometry was done using a BD-LSRFortessa flow cytometer (BD) and the results were displayed in the FACSDiva software. Cells and organoids were imaged using an EVOS FL microscope (Thermo Scientific) with brightfield, GFP and TexasRed filters.
Data analysis	Confocal images were processed and analyzed using the LAS software (Leica) and ImageJ (v1.52). Flow cytometry data analysis were performed using FlowJo (v10.7.1, BD).

For manuscripts utilizing custom algorithms or software that are central to the research but not yet described in published literature, software must be made available to editors and reviewers. We strongly encourage code deposition in a community repository (e.g. GitHub). See the Nature Portfolio guidelines for submitting code & software for further information.

Data

Policy information about availability of data

All manuscripts must include a data availability statement. This statement should provide the following information, where applicable:

- Accession codes, unique identifiers, or web links for publicly available datasets
- A description of any restrictions on data availability
- For clinical datasets or third party data, please ensure that the statement adheres to our policy

Source data for all figures will be included in the paper.

Research involving human participants, their data, or biological material

Policy information about studies with human participants or human data. See also policy information about sex, gender (identity/presentation), and sexual orientation and race, ethnicity and racism.

Reporting on sex and gender	This study did not include human participants.
Reporting on race, ethnicity, or other socially relevant groupings	This study did not include human participants.
Population characteristics	This study did not include human participants.
Recruitment	This study did not include human participants.
Ethics oversight	This study did not include human participants.

Note that full information on the approval of the study protocol must also be provided in the manuscript.

Field-specific reporting

Please select the one below that is the best fit for your research. If you are not sure, read the appropriate sections before making your selection.

☒ Life sciences ☐ Behavioural & social sciences ☐ Ecological, evolutionary & environmental sciences

For a reference copy of the document with all sections, see nature.com/documents/nr-reporting-summary-flat.pdf

Life sciences study design

All studies must disclose on these points even when the disclosure is negative.

Sample size	No statistical methods were used to predetermine sample size for the experiments and clone size quantification. The determination for the sample size was based on known literatures (n=3-6) (DOI: 10.1126/science.1196236. and DOI: 10.1016/j.cell.2010.09.016. and doi.org/10.1038/s41586-021-03605-0)
Data exclusions	No data were excluded from the analysis in this study.
Replication	Experiments were carried out with at least 2 independent animals. Transfection experiments were repeated at least 2 independent times, where the representative data were shown.
Randomization	Based on the genotype, cohorts including both sexes were chosen and randomly distributed into different experimental time points.
Blinding	Blinding was not carried out. The genotype of the mice needed to be determined before each experiment. The DNA materials used in transfection needed to be verified and confirmed prior the experiments.

Reporting for specific materials, systems and methods

We require information from authors about some types of materials, experimental systems and methods used in many studies. Here, indicate whether each material, system or method listed is relevant to your study. If you are not sure if a list item applies to your research, read the appropriate section before selecting a response.

Materials & experimental systems

n/a	Involved in the study	
☐	☒	Antibodies
☐	☒	Eukaryotic cell lines
☒	☐	Palaeontology and archaeology
☐	☒	Animals and other organisms
☒	☐	Clinical data
☒	☐	Dual use research of concern
☒	☐	Plants

Methods

n/a	Involved in the study	
☒	☐	ChIP-seq
☐	☒	Flow cytometry
☒	☐	MRI-based neuroimaging

Antibodies

Antibodies used	Rat monoclonal anti-SOX2 Invitrogen Cat# 14-9811-82 1:200

Antibodies used	Mouse monoclonal anti-β-Catenin (L54E2) (Alexa Fluor 647 conjugate) Cat# 4627 1:200<br>Donkey polyclonal anti-rat secondary antibody Alex Fluor Plus 647 Cat# A48272 1:500
Validation	Rat monoclonal anti-SOX2 Invitrogen Cat# 14-9811-82, the antibody was validated in knockdown experiments. https://www.thermofisher.com/antibody/product/SOX2-Antibody-clone-Btjce-Monoclonal/14-9811-82<br><br>Mouse monoclonal anti-β-Catenin (L54E2) (Alexa Fluor 647 conjugate) Cat# 4627, the antibody was validated in knockout experiments in previously published articles (doi.org/10.1038/s12276-022-00891-0). https://www.cellsignal.com/products/antibody-conjugates/b-catenin-l54e2-mouse-mab-alexa-fluor-647-conjugate/4627

Eukaryotic cell lines

Policy information about cell lines and Sex and Gender in Research

Cell line source(s)	Hek293T cells (ATCC® CRL-11268™); mouse AN3-12 ES cells (doi.org/10.1038/nature24027); Mouse adult esophageal organoids, isolated, established and maintained in this study.
Authentication	Cells were not authenticated with molecular or expression analysis, but were verified with the expected morphologies.
Mycoplasma contamination	Cells were routinely tested for mycoplasma during cultures and were confirmed negative.
Commonly misidentified lines (See ICLAC register)	There were no commonly misidentified lines in this study to our knowledge.

Animals and other research organisms

Policy information about studies involving animals; ARRIVE guidelines recommended for reporting animal research, and Sex and Gender in Research

Laboratory animals	Vil-CreERT2 mouse (JAX, 020282), RCE:FRT mouse (#032038-JAX) were obtained from Jackson Laboratory. Sox2-SCONFRT conditional knockout mouse was generated via zygote injection (doi.org/10.1038/s12276-022-00891-0). The Red2Flpe and Apc-FRT mice were generated in this study. Experiments were performed in adult mice of 8-20 week-old mice. All animals were kept in specific pathogen-free animal facilities, under light/dark cycle of 14 h:10 h (light summertime, 07:00–21:00; light wintertime, 06:00–20:00).
Wild animals	No wild animals were used in this study.
Reporting on sex	Sex was not a consideration in this study. All animal experiments consisted of both sexes.
Field-collected samples	There were no field-collected samples in this study.
Ethics oversight	All animal experiments were performed according to the guidelines of the Austrian Animal Experiments Act; with valid project licenses approved by the Austrian Federal Ministry of Education, Science and Research; and monitored by the institutional IMBA Ethics and Biosafety department.

Note that full information on the approval of the study protocol must also be provided in the manuscript.

Flow Cytometry

Plots

Confirm that:

☒ The axis labels state the marker and fluorochrome used (e.g. CD4-FITC).

☒ The axis scales are clearly visible. Include numbers along axes only for bottom left plot of group (a 'group' is an analysis of identical markers).

☒ All plots are contour plots with outliers or pseudocolor plots.

☒ A numerical value for number of cells or percentage (with statistics) is provided.

Methodology

Sample preparation	Cultured cells were washed once with 1x PBS and incubated in 1x trypsin-EDTA at 37 degrees Celsius until the majority of the cells detached. Culture media, which contains FBS, was used to quench the trypsin enzymatic activity and cells were dissociated into single cells. The suspension was collected and spun down at 200x g for 3 minutes and the clear supernatant was removed by aspiration. The cell pellet was resuspended in culture media and passed through a 40 µm filter prior the flow cytometry analysis.
Instrument	BD-LSRFortessa flow cytometer (BD) equipped with the FACSDivaTM software (BD)
Software	The flow cytometry data were analyzed with Flowjo v10.7.1

Cell population abundance	Population abundance in the sorted cultured cells were not determined, as the cells were subjected to further cultures and flow cytometry analysis.
Gating strategy	To determine the suitable positive gating strategy, negative controls which were untransfected or transfected with only mCherry were used as baseline comparisons.

☒ Tick this box to confirm that a figure exemplifying the gating strategy is provided in the Supplementary Information.
